# Augmentation of Tonic GABA_A_ Inhibition in Absence Epilepsy: Therapeutic Value of Inverse Agonists at Extrasynaptic GABA_A_ Receptors

**DOI:** 10.1155/2011/790590

**Published:** 2011-09-05

**Authors:** Adam C. Errington, David W. Cope, Vincenzo Crunelli

**Affiliations:** ^1^School of Biosciences, Cardiff University, Museum Avenue, Cardiff CF10 3US, UK; ^2^Neuroscience Division, School of Biosciences, Cardiff University, Museum Avenue, Cardiff CF10 3AX, UK

## Abstract

It is well established that impaired GABAergic inhibition within neuronal networks can lead to hypersynchronous firing patterns that are the typical cellular hallmark of convulsive epileptic seizures. However, recent findings have highlighted that a pathological enhancement of GABAergic signalling within thalamocortical circuits is a necessary and sufficient condition for nonconvulsive typical absence seizure genesis. In particular, increased activation of extrasynaptic GABA_A_ receptors (eGABA_A_R) and augmented “tonic” GABA_A_ inhibition in thalamocortical neurons have been demonstrated across a range of genetic and pharmacological models of absence epilepsy. Moreover, evidence from monogenic mouse models (stargazer/lethargic) and the polygenic Genetic Absence Epilepsy Rats from Strasbourg (GAERS) indicate that the mechanism underlying eGABA_A_R gain of function is nonneuronal in nature and results from a deficiency in astrocytic GABA uptake through the GAT-1 transporter. These results challenge the existing theory that typical absence seizures are underpinned by a widespread loss of GABAergic function in thalamocortical circuits and illustrate a vital role for astrocytes in the pathology of typical absence epilepsy. Moreover, they explain why pharmacological agents that enhance GABA receptor function can initiate or exacerbate absence seizures and suggest a potential therapeutic role for inverse agonists at eGABA_A_Rs in absence epilepsy.

## 1. Introduction

Typical absence epilepsy is characterised by the regular occurrence of nonconvulsive seizures that result in periods of sudden and brief (average *≈*10 seconds, range *≈*4–40 seconds) loss of consciousness. In the electroencephalogram (EEG), human absence seizures are typified by the appearance of generalized, synchronous, and bilateral “spike (or polyspike) and slow wave discharges” (SWD) occurring at frequencies between 2.5–4 Hz [1, 2]. Although typical absence seizures are significant clinical features of many generalized idiopathic epilepsies (IGEs), as defined by the classification of the International League Against Epilepsy (ILAE) [3], they are the only neurological symptom presented in childhood absence epilepsy (CAE). CAE has an annual incidence of approximately 2–8 per 100,000 children under 16 years of age, with seizure onset typically occurring between 3 and 8 years of age and seizure frequency often as high as several hundred events per day [2]. Absence seizures associated with CAE are not triggered by visual or other sensory stimuli and are not usually associated with neurometabolic or neurophysiological deficits, a factor which is thought to contribute to ~70% spontaneous remission rates in adolescence [2, 4]. Nonetheless, in this pure absence epilepsy phenotype, there is a consensus, based upon older invasive studies and more recent imaging investigations, that seizure genesis and propagation occur as a result of aberrant electrical activity in reciprocally connected thalamic and cortical regions (i.e., thalamocortical circuits) without significant involvement of other brain areas including hippocampus and limbic regions which are often associated with convulsive seizures [5–9]. In fact, recent observations in humans suggest that seizure genesis occurs due to paroxysmal activation of discrete frontal and parietal cortical territories prior to spread into other cortical and thalamic regions [5–8]. This review will, therefore, focus on the key cellular elements of thalamocortical circuits and in particular upon thalamocortical neurons. 

*γ*-aminobutyric acid (GABA) is the principal inhibitory neurotransmitter in the brain, and its actions are mediated largely by a family of ubiquitously expressed ligand-gated ion channels known as GABA_A_ receptors [10]. GABA_A_ receptors are pentameric assemblies comprising several distinct subunits which open upon GABA binding leading to an increase in membrane permeability to both chloride and bicarbonate ions [11]. Typically this occurs when GABA is released from presynaptic terminals causing a transient rise in GABA concentration within the synaptic cleft and activation of postsynaptic receptors. The resulting brief change in membrane conductance underlies “phasic” GABA_A_ergic inhibition and generation of the “classical” inhibitory postsynaptic potential (IPSP). However, it has come to light relatively recently that GABA_A_ receptor activation can occur in a much more spatially and temporally diffuse manner [10]. It has been demonstrated in several brain regions including the cerebellum [12], hippocampus [13], and thalamus [14–16] that very low (nM) concentrations of GABA, which are found in the extracellular space, can persistently activate a population of nonsynaptic GABA_A_ receptors resulting in a “tonic” increase in membrane conductance. These peri- or extrasynaptic GABA_A_ receptors (eGABA_A_Rs) differ from their synaptic counterparts in having a significantly higher affinity for GABA as well as markedly slower rates of desensitization [10, 17–19] although it has been recently demonstrated in the visual thalamus that significant desensitization of eGABA_A_Rs can occur at ambient GABA concentrations [20]. The divergence in the properties of synaptic GABA_A_Rs versus eGABA_A_Rs is conferred by receptor subunit composition, in particular, the inclusion of the *δ* subunit in the case of dentate gyrus granule cells (DGCs), cerebellar granule cells (CGCs), thalamocortical neurons and some cortical neurons [13–16, 21, 22], and *α*_5_ subunits in CA1 and CA3 hippocampal pyramidal cells [23–25]. Nineteen GABA_A_ receptor subunits have been cloned from the mammalian CNS (*α*_(1–6)_, *β*_(1–3)_, *γ*_(1–3)_, *δ*, *ε*, *θ*, *π*, *ρ*_(1–3)_) offering the potential for an enormous heterogeneity in GABA_A_ receptor assembly. In reality however, only about twenty to thirty of the potential combinations have been shown to exist in the brain. The most commonly expressed subunit combination is *α*_1_, *β*_2_, *γ*_2_ (with stoichiometry of 2*α* and 2*β* subunits and a single *γ* subunit [26, 27]) whilst other common arrangements include *α*_2_ *β*_3_ *γ*_2_ and *α*_3_ *β*_3_ *γ*_2_. Significantly, light microscopic immunofluorescence and EM immunogold methods have established that the postsynaptic densities of GABAergic synapses are highly enriched with receptors including *α*_(1–3)_, *α*_6_, *β*_(2-3)_, and *γ*_2_ subunits [28, 29] suggesting that these subunits form the GABA_A_ receptors responsible for classical “phasic” inhibition. However, in contrast to the aforementioned subunits which are enriched in the postsynaptic density but also abundant at extrasynaptic locations [30, 31], some GABA_A_ receptor subunits, especially *δ*, are not found in the synapse and are exclusively peri- or extrasynaptically located [21]. Extrasynaptic receptors containing the *δ* subunit are commonly found to coassemble with *α*_4_ or *α*_6_ subunits (*α*_4_/*α*_6_ *β*_X_ *δ*) whilst *α*_5_-containing receptors are also mostly extrasynaptic despite usually containing the typically synaptically located *γ*_2_ subunit (*α*_5_ *β*_X_ *γ*_2_). A recent study by Kasugai et al. [32] has demonstrated the presence of *α*_1_ and *α*_2_ subunits as well as *β*_3_ subunits at extrasynaptic locations on the soma of CA1 pyramidal neurons suggesting these subunits may also contribute to eGABA_A_R signalling and perhaps confer specific pharmacological properties. 

Thalamocortical (TC) neurons of the dorsal lateral geniculate nucleus (dLGN, visual thalamus) [14], the ventrobasal nuclei (VB, somatosensory thalamus) [14–16], and the medial geniculate body (MGB, auditory thalamus) [33] of rodents have been demonstrated *in vitro *to have robust GABAergic tonic currents. In voltage-clamped TC neurons, application of the GABA_A_ receptor antagonist SR-95531 not only completely blocks the phasic inhibitory postsynaptic currents (IPSCs) but also produces a reduction in input conductance accompanied by a decrease in current variance that is indicative of block of tonically active eGABA_A_Rs. In the thalamus, it has been estimated that approximately 80–90% of total GABA_A_ receptor-mediated inhibition occurs through tonic currents resulting from activation of extrasynaptic GABA_A_Rs [14, 16]. In fact, it has been suggested that tonic conductance in TC neurons (when normalized to whole cell capacitance) may be larger than in other regions expressing eGABA_A_Rs including the cerebellum and dentate gyrus [16]. In all of the previously described thalamic nuclei, there is a high expression of the GABA_A_ receptor *δ*-subunit [22, 33–35], and several studies have shown, using selective pharmacological agents [14–16], *δ*-subunit knock-out (*δ* ^−/−^) [36] and *α*_4_-subunit knock-out (*α*_4_^−/−^) [37] mice that the thalamic tonic current is mediated largely by *α*_4_ *β*_2_ *δ* subunit-containing receptors. In particular, eGABA_A_Rs in the thalamic nuclei are highly sensitive to the potent and selective activator of *α*_4_-*δ*-containing [38] receptors 4,5,6,7-tetrahydroisoxazolo[5,4-c]-pyridin-3-ol (THIP, Gaboxadol) [14–16, 37] as well as ethanol [39], taurine [40], and the anaesthetic isoflurane [41], all of which act to enhance tonic inhibition. Conversely, the *α*_1_-selective agent zolpidem and the nonselective benzodiazepine midazolam increase the decay time of sIPSCs in VB neurons without effects on tonic currents and the inverse agonist Ro 15-4513, a potent activator of *α*_4_-*γ*_2_ subunit-containing receptors [38], also had no effect of tonic current in VB [15]. Functionally, eGABA_A_Rs in the thalamus have been suggested to play a role in switching the behavioural state-dependent TC neuron firing modes [14] and modulating the temporal precision of rebound low-threshold Ca^2+^ spikes (LTS) [34]. Furthermore, tonic inhibition in TC neurons is likely to play a significant role in the modulation of slow wave sleep (SWS) activity given the integral role of TC neurons in generating low-frequency (<4 Hz) oscillations in corticothalamic circuits [14, 16]. However, the potential importance of eGABA_A_Rs in pathological seizure activity associated with typical absence epilepsy has only recently been elucidated.

## 2. Enhanced Tonic GABA_A_ Inhibition in Thalamocortical Neurons of Genetic Absence Epilepsy Models

**Figure 1:**
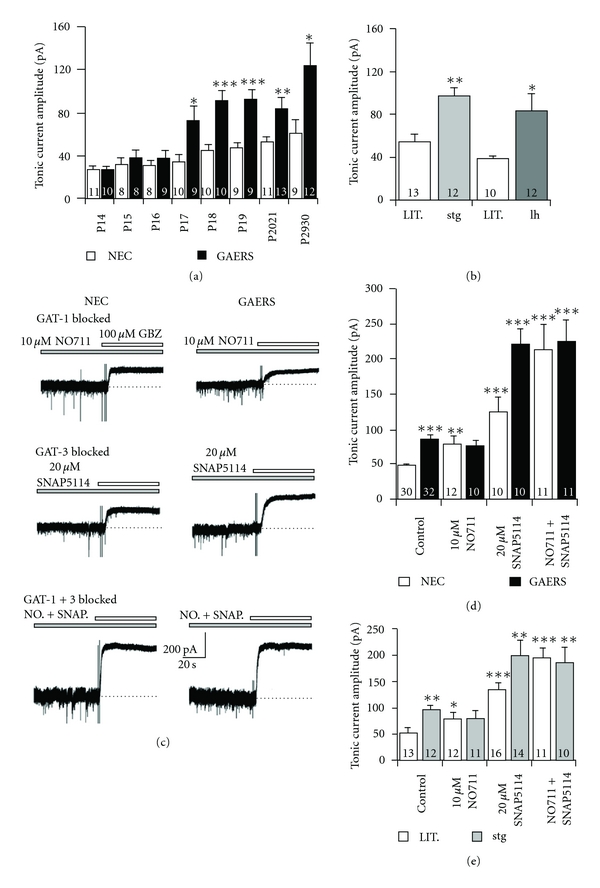
GAT-1 transporter dysfunction produces enhanced tonic GABA_A_ currents in VB TC neurons in animal models of absence epilepsy. (a) The developmental profile of enhanced thalamic tonic current observed in GAERS animals compared to NEC. At P17 (prior to seizure onset), a significant increase in current amplitude is observed in the epileptic animal that remains elevated up to seizure onset (P30). (b) Tonic GABA_A_ currents in VB TC neurons of monogenic stargazer (stg) and lethargic (l hour) mice are significantly greater than nonepileptic littermates after seizure onset. (c) Block of GAT-1 using NO711 in NEC animals elevates tonic current amplitude to levels similar to those observed in GAERS animals. No further enhancement of tonic current in GAERS is observed when GAT-1 is blocked. Block of GAT-3 produces significant increases in tonic current in both NEC and GAERS animals although the increase is smaller in NEC where GAT-1 remains functional. Simultaneous block of GAT-1 and GAT-3 results in very large tonic currents in both GAERS and NEC animals, which are not significantly different from each other. (d) Graph summarising the experiments depicted in (c). (e) Graph depicting the same series of experiments performed in stargazer mice illustrating the similar effects in both models. *P < 0.05, **P < 0.01, ***P < 0.001. Number of recorded neurons for each condition is indicated in bars. (a–e) reproduced from [34].

It has been demonstrated *in vitro* using several different genetic animal models of absence seizures that the tonic GABA_A_ current in TC neurons of the VB thalamus is enhanced in animals displaying an epileptic phenotype compared to their respective nonepileptic control animals (Figure 1) [36]. This was first shown in the polygenic GAERS model but has subsequently also been demonstrated for various mice models with known, but divergent, spontaneous monogenic mutations, including stargazer and lethargic mice. In GAERS animals, there is a clear developmental profile for this increased GABAergic function (Figure 1(a)). Up to postnatal day sixteen, the tonic current in VB of GAERS is similar to that of the nonepileptic control (NEC) strain. However, in the 24 hour period between the postnatal day 16-17, there is a significant (almost doubling) increase in the amplitude of the tonic current in VB TC neurons of the epileptic animals [36] that remains elevated well past the time of seizure onset (around the postnatal day 30 in this strain). These data suggest that, rather than occurring as a consequence of seizure onset, the pathological enhancement of tonic GABA inhibition during development in GAERS may be proepileptogenic. Moreover, despite the full developmental profile for the monogenic lethargic and stargazer mice being unknown, it is clear in these models that a significant enhancement of tonic current in TC neurons is present after seizure onset, (Figure 1(b)) [36]. In contrast, no tonic GABA_A_ current is detected in the GABAergic NRT neurons of GAERS or NEC animals (unpublished observation) as is indeed the case in normal Wistar rats [14].

The pathological augmentation of tonic GABA_A_ currents in TC neurons of genetic absence models is, however, not due to increased vesicular GABA release, overexpression of *δ*-subunit containing eGABA_A_Rs, or misexpression of synaptic GABA_A_Rs but results from a dysfunction of GABA re-uptake by the transporter GAT-1 [36]. In fact, despite being far less abundant in the thalamus than GAT-3 [42], GAT-1 appears to play a major role in the regulation of extrasynaptic GABA concentration and activation of eGABA_A_Rs [36]. In acute brain slices prepared from both GAERS and stargazer animals, block of GAT-1 using the specific antagonist NO711 produced no effect upon the magnitude of tonic current observed in VB TC neurons, (Figures 1(c), 1(d), and 1(e)). In marked contrast, the block of GAT-1 in neurons of nonepileptic mice and rats facilitated a significant enhancement of tonic current that reached levels similar to those found in neurons from epileptic animals, (Figures 1(c), 1(d), and 1(e)). Furthermore, in nonepileptic animals, blockade of GAT-3 using SNAP5114 resulted in an increase in tonic current that was significantly less than that observed in GAERS or stargazer suggesting that the ability of GAT-1 to compensate for the loss of GAT-3 is erased in the epileptic strains, (Figures 1(c), 1(d), and 1(e)). These findings are made all the more significant by the fact that expression of both GAT-1 and GAT-3 in the thalamus appears to occur exclusively in nonneuronal cells, specifically astrocytes [42, 43]. A malfunction in GAT-1 also underlies the increased tonic GABA_A_ current in TC neurons of lethargic mice [36]. In contrast to GAERS and stargazer mice; however, the action of this transporter is not inhibited in lethargic mice but appears to be reversed. These data expand upon previous findings that demonstrated a reduction in GABA uptake by GAT-1 [44] and increased levels of extracellular GABA [45] in the VB thalamus of GAERS compared to NEC. Moreover, NO711 increases tonic GABA_A_ current by a similar amount in dentate gyrus granule cells of GAERS and NEC [36], indicating that GAT-1 activity is not compromised in a brain area that does not participate in the generation of typical absence seizures and where the distribution of this transporter is primarily neuronal. Indeed, the basal tonic current of dentate gyrus granule cells is not different between GAERS and NEC [36], and in stargazer mice, tonic current in both DGCs and CGCs is actually reduced compared to WT littermates [46]. Interestingly, it has been demonstrated previously in CGCs of GABA_A_R *α*_1_ subunit knock-out (*α*_1_^−/−^) mice that tonic currents in these neurons are also enhanced via a reduction of GAT activity that is not due to reduction in GAT-1 or GAT-3 expression or increased expression of either *α*_6_ or *δ* subunit-containing receptors [47].

In summary; therefore, genetic models of typical absence seizures (i.e., GAERS, stargazer, and lethargic mice) show a brain region-specific enhancement of tonic GABA_A_ current, which in TC neurons is due to increased extracellular GABA level that in turn results from a malfunction in GABA uptake by astrocytic GAT-1.

## 3. Pharmacological Models of Typical Absence Epilepsy and the Role of GABA_B_ Receptors

**Figure 2:**
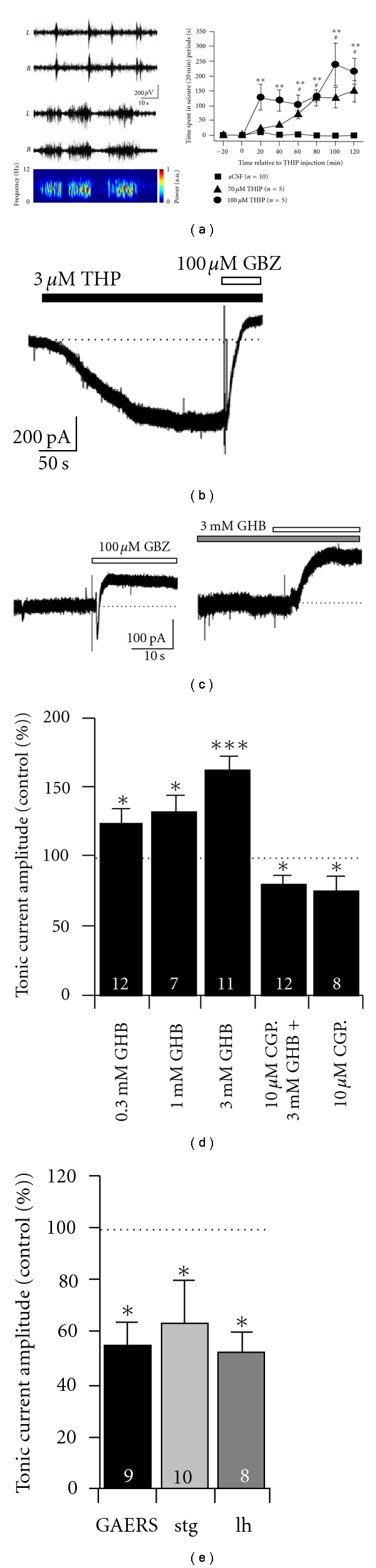
GHB and THIP enhance tonic GABA_A_ currents in VB thalamus *in vitro* and induce SWDs *in vivo*. (a) Examples of SWDs in bilateral EEG traces recorded from Wistar rats following selective activation of eGABA_A_Rs by intrathalamic application of THIP via microdialysis (100 *μ*M). The top trace shows seizures occurring in the first hour after THIP administration and the bottom the second hour. The spectrogram (corresponding to the right hemisphere of the lower traces) clearly shows an increase in oscillatory power in the 5–7 Hz range typical of SWDs in rats. The graph (right) summarises the concentration-dependent emergence of SWDs after THIP application as the total time spent in seizure during 20 minutes bins. (b) THIP produces robust enhancement of tonic GABA_A_ currents in VB TC-neurons in acute brain slices *in vitro*. (c) GHB produces increased tonic current in VB TC neurons. (d) Graph summarising the concentration-dependent enhancement of thalamic tonic currents by GHB and the blocking effect of the GABA_B_R antagonist CGP55845. (e) Block of GABA_B_Rs by CGP55845 produces a reduction of tonic GABA_A_ currents in VB TC neurons of epileptic GAERS, stargazer, and lethargic mice. *P < 0.05, **P < 0.01, ***P < 0.001. Number of recorded neurons for each condition is indicated inset into bars. (a–e) reproduced from reference [34].

As well as resulting from genetic modifications, SWDs can be generated in genetically “normal” animals through administration of various pharmacological agents. The best-established pharmacological model of typical absence seizures is achieved by the systemic administration of *γ*-hydroxybutyric acid (GHB) [48–50]. However, it has been known for some time that systemic administration of THIP, a selective agonist at *δ* subunit-containing extrasynaptic GABA_A_Rs, also elicits SWDs in normal animals, (Figure 2(a)) [51]. In the context of the involvement of enhanced thalamic tonic GABA_A_ inhibition in several genetic models of absence epilepsy, the pharmacological induction of seizures by THIP becomes more readily explainable. This is because, as previously disclosed, THIP can potently enhance tonic GABA_A_ currents of TC neurons in nonepileptic rats, (Figure 2(b)), [36] and mice [15, 16], thus mimicking the enhanced thalamic tonic inhibition observed in genetic models. On the other hand, the effects of GHB, which does not bind to GABA_A_Rs and is believed to elicit absence seizures by activation of GABA_B_Rs [50], become more difficult to interpret in light of the apparent necessity for enhanced eGABA_A_R signalling during SWDs. However, it has now been demonstrated in brain slices of Wistar rats that GHB enhances tonic GABA_A_ currents in TC neurons, (Figure 2(c)) [36]. The effects on tonic GABAergic inhibition *in vitro* are dose dependent with concentrations used reflecting those that are required to elicit absence seizures *in vivo*, (Figure 2(d)) [52]. Moreover, the effects of GHB are not due to nonspecific binding interactions since the GHB-mediated enhancement of tonic current is negated by the GABA_B_R antagonist CGP55845, (Figure 2(d)). In fact, application of CGP55845 alone significantly reduces the tonic GABA_A_ current amplitude in TC neurons of Wistar rats to 74% of the control values, indicating that facilitation of extrasynaptic GABA_A_Rs by GABA_B_Rs contributes approximately one quarter of the tonic GABA_A_ current in normal rats. Importantly, CGP55845 also reduces the tonic current in GAERS, stargazer, and lethargic mice to about 55, 65, and 57% of control, (Figure 2(e)), respectively, [36] suggesting that facilitation of extrasynaptic GABA_A_R function by GABA_B_R activation contributes up to half of the pathologically enhanced tonic current in these genetic models.

**Figure 3:**
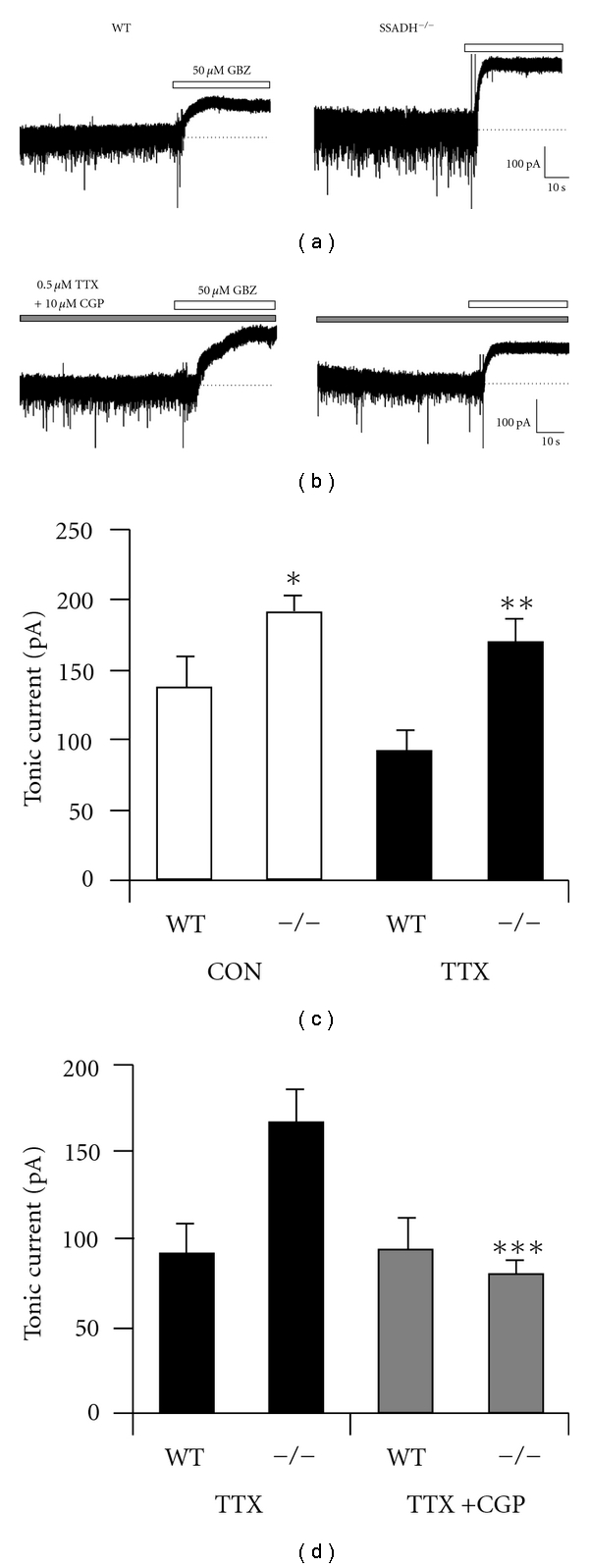
SSADH-deficient mice have enhanced tonic GABA_A_ currents in VB TC neurons. (a) and (c) VB TC neurons from SSADH^−/−^ mice display enhanced tonic GABA_A_ currents compared with their WT control littermates. (b) and (d) In TTX, tonic current amplitudes in both SSADH^−/−^ and WT mice are reduced compared to control conditions. CGP55845 reduces the amplitude of the tonic GABA_A_ current observed in SSADH^−/−^ to a similar level found in WT mice. (a–d) reproduced from reference [58].

This GHB-mediated enhancement of thalamic tonic currents is fascinating in the context of another genetic disorder related to GABAergic system function. Succinic semialdehyde dehydrogenase (SSADH) deficiency is an autosomal recessively inherited disorder that results in loss of activity in SSADH (an enzyme responsible for metabolism of GABA), reduced GABA breakdown, and excessive accumulation of both GABA and GHB in the cerebrospinal fluid [53, 54]. Clinical symptoms are varied but include delayed intellectual, speech and language development, ataxia and, significantly, generalised absence seizures [55–57]. Using a recently developed SSADH knock-out (SSADH^−/−^) mouse [53, 58, 59], we have been able to demonstrate that in these animals, which replicate the epileptic phenotype displayed in humans with SSADH deficiency, there is a significant enhancement of tonic GABA_A_ currents in TC neurons compared to their WT counterparts, Figures 3(a) and 3(c) [60]. Moreover, as previously described for other genetic models of absence seizures, a large proportion of the enhanced tonic current is sensitive to block by GABA_B_R antagonists further supporting the role of these metabotropic receptors in the pathology of absence seizures, (Figures 3(b) and 3(d)) [60].

In summary; therefore, a GAT-1 malfunction in thalamic astrocytes of mouse and rat genetic models leads to an increase in ambient GABA in the sensory thalamus, which in turn elicits an enhancement in tonic GABA_A_ inhibition through direct activation of extrasynaptic GABA_A_Rs and indirect facilitation of extrasynaptic GABA_A_Rs via activation of GABA_B_Rs. 

## 4. Enhanced Tonic GABA_A_ Inhibition of TC Neurons Is Necessary and Sufficient for Typical Absence Seizure Generation

As previously described, SWDs of typical absence epilepsy appear to be initiated in deep layers (V/VI) of the cortex where intracellular recordings show rhythmic paroxysmal depolarisations occurring in phase with the EEG spike [61–63]. The action potentials associated with these synchronous depolarisations in turn provide strong rhythmic input to thalamic nuclei. In NRT neurons *in vivo*, the strong converging corticothalamic input that result from cortical volleys during SWDs produces bursts of excitatory postsynaptic potentials (EPSPs) that trigger T-type Ca^2+^-channel-mediated LTS and bursts of action potentials. In contrast, TC neurons receive both monosynaptic excitation directly from corticothalamic inputs and disynaptic inhibition via the NRT. *In vivo *intracellular recordings made in GAERS have shown that during ictal activity TC neurons typically receive sequences of one EPSP plus four to six IPSPs arriving in phase with each EEG spike and that action potential firing is rare [62, 64]. This is likely due to the much stronger corticothalamic excitatory inputs into NRT neurons compared to TC neurons [65] and the robust nature of the LTS-driven action potential bursts of NRT neurons [62, 64]. Thus, it is highly probable although it remains to be directly demonstrated that strong GABAergic input into TC neurons during SWDs produces activation of eGABA_A_Rs and that the corresponding increase in tonic current contributes to the observed downregulation of TC neuron output during ictal activity.

**Figure 4:**
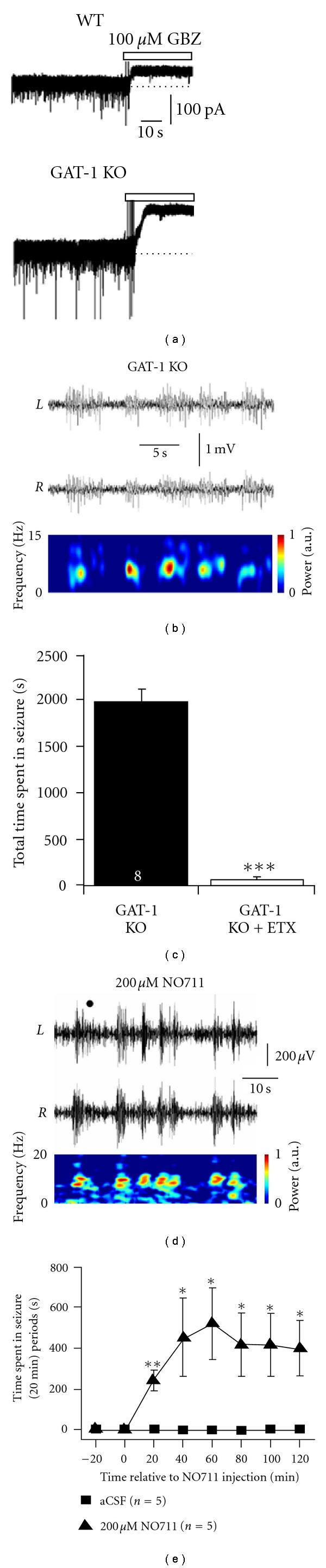
Loss of functional GAT-1 in TC neurons leads to SWDs. (a) In adult (P68-74) GAT-1^−/−^ mice, tonic GABA_A_ currents in VB TC neurons are significantly larger than in WT littermates. (b) Simultaneous bilateral EEG traces reveal that adult GAT-1^−/−^ mice also display SWDs (WT animals did not display SWDs—data not shown). The spectrogram at the bottom corresponds to the EEG signal from the right (R) hemisphere. (c) Treatment of GAT-1^−/−^ mice with the anti-absence drug ethosuximide (200 mg per kg body weight i.p.) significantly reduces the total time spent in seizures. (d) Bilateral EEG traces from a normal Wistar rat following intrathalamic administration by reverse microdialysis of 200 *μ*M of the selective GAT-1 blocker NO711 (spectrogram of the L trace is illustrated below). (e) Time course of the induction of SWDs by intrathalamic administration of NO711. (a–e) reproduced from [34].

**Figure 5:**
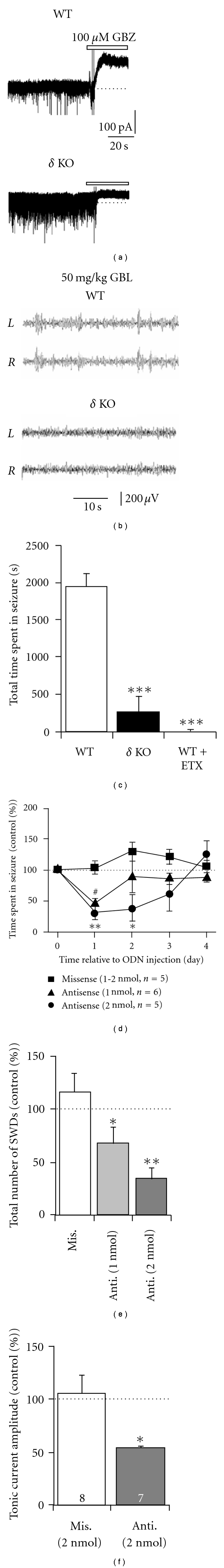
*δ* subunit containing eGABA_A_RS in thalamocortical neurons are crucial for expression of SWDs. (a) Tonic GABA_A_ currents are nearly completely ablated in VB TC neurons from *δ* ^−/−^ mice. In comparison, WT mice display robust tonic currents as revealed by focal application of GBZ to the recording chamber. (b) Bilateral EEG traces demonstrating that GBL (the GHB prodrug) induces SWDs in WT littermates but not in *δ* ^−/−^ mice. (c) Ethosuximide-sensitive SWDs that are observed in GBL-injected WT mice are significantly reduced in *δ* ^−/−^ mice. Graph summarises the total time spent in seizure. (d) Intrathalamic injection of *δ* subunit-specific antisense oligodeoxynucleotides (ODN) produced a significant reduction in time spent in seizure in GAERS for two days post injection. In contrast sham injection of a missense ODN had no significant effect on the occurrence of SWDs. (e) Graph summarising the effect of ODN injection into VB thalamus upon seizure number normalized to preinjection control values. (f) Graph summarising the effect of anti- and missense ODN injection into VB thalamus of GAERS animals upon tonic current amplitude measured in vitro. Acute brain slices were prepared 1 day after intrathalamic injections were administered. (a–f) reproduced from [34].

To assess the impact that the enhanced tonic GABA_A_ current of TC neurons might have in the expression of absence seizures, experiments in freely moving animals are required. Under these conditions, both the behavioural and EEG components of the seizures can be assessed, and data are not confounded by the concomitant use of anaesthetics and/or analgesics. Thus, unrestrained GAT-1 KO mice (GAT-1^−/−^), which have not undergone any pharmacological treatment and whose TC neurons display enhanced tonic GABA_A_ currents *in vitro* express ethosuximide-sensitive typical absence seizures (Figures 4(a), 4(b) and 4(c)) [36]. Furthermore, the direct injection of the selective GAT-1 blocker NO-711 into the VB by reverse microdialysis initiates ethosuximide-sensitive typical absence seizures in previously nonepileptic Wistar rats (Figures 4(d) and 4(e)) [36]. On the other hand, in *δ* ^−/−^ mice, which exhibit a nearly ablated tonic GABA_A_ inhibition in TC neurons (Figure 5(a)), systemic administration of GHB fails to induce absence seizures (Figures 5(b) and 5(c)) [36]. Intrathalamic injection of a *δ* subunit-specific antisense oligodeoxynucleotide in GAERS strongly decreases both the tonic GABA_A_ current and spontaneous seizures 1-2 days after injection, whereas a missense oligodeoxynucleotide has no effect (Figures 5(d), 5(e), and 5(f)) [36]. Finally, intrathalamic administration of THIP in normal Wistar rats elicits absence seizures in a concentration-dependent manner, which as expected are blocked by systemic administration of ethosuximide [36]. Taken together, these data show that enhanced tonic GABA_A_ inhibition in TC neurons is both necessary and sufficient for the generation of typical absence seizures. 

## 5. Conclusions and Future Perspectives

 Augmented tonic GABA_A_ inhibition in TC neurons represents the first potential molecular mechanism that is common to both well-established pharmacological and genetic models of typical absence seizures. Despite having a range of divergent genetic mutations, GAERS (polygenic), stargazer (Ca^2+^ channel *γ*_2_ subunit, TARP-*γ*_2_), lethargic (Ca^2+^ channel *β*_4_ subunit), SSADH^−/−^ and GAT-1^−/−^ mice all display SWDs characteristic of typical absence epilepsy, whereas in *δ* ^−/−^ mice drugs that commonly produce SWDs are ineffective. Importantly, because powerful GABA_A_ IPSPs can be recorded in the vast majority of TC neurons during absence seizures *in vivo* [64, 66], these findings also indicate that model systems that aim to reproduce typical absence seizures by blocking GABA_A_Rs of TC neurons are inherently flawed.

The discovery of a malfunction in GAT-1 as the underlying abnormality that produces increased tonic GABA_A_ inhibition in TC neurons of genetic absence models shifts the emphasis from a neuronal to an astrocytic aetiology for this type of nonconvulsive epilepsy. Impaired GAT-1 activity in GAERS is not caused by decreased thalamic or cortical expression of GAT-1 mRNA or protein levels. Also, no genetic variants are present in GAT-1 cDNA from GAERS, stargazer, or lethargic mice nor are the mutations responsible for absence seizures in stargazer and lethargic mice present in GAERS. Future studies, therefore, may investigate whether GAT-1 is unable to reach the outer astrocytic membrane and/or whether there are abnormalities in its phosphorylation processes. 

Experimental typical absence seizures can be elicited or aggravated by selective GABA_B_R agonists and can be blocked by selective GABA_B_R antagonists, applied either systemically or intrathalamically. Because about 50% of the tonic GABA_A_ current observed in TC neurons of GAERS, stargazer, and lethargic and SSADH^−/−^ mice is abolished by a GABA_B_ antagonist [36, 60], the behavioural and EEG effects of selective GABA_B_ drugs on typical absence seizures can no longer be simply explained by the ability of these drugs to affect GABA_B_ IPSPs and/or presynaptic GABA_B_Rs but should also take into account the positive modulation by GABA_B_Rs of the tonic GABA_A_ inhibition in TC neurons.

From a clinical perspective, it is important to stress that all the results reviewed above provide a mechanistic explanation for the aggravation of absence seizures that is observed in humans and experimental animals following either systemic or intrathalamic administration of drugs that increase GABA levels, including tiagabine, a GABA uptake blocker, and vigabatrin, a GABA transaminase blocker [67–70]. Thus, the classical approach of treating seizures by increasing inhibition through positive modulation of GABA-ergic neurotransmission is particularly ineffective in absence epilepsy. In this circumstance, a selective *reduction* of tonic GABA_A_ inhibition in thalamic neurons presents perhaps the best possible therapeutic intervention. Intriguingly, a recent study demonstrated that excessive tonic GABA_A_ergic inhibition is also a feature of cortical neurons surrounding the infarct site (peri-infarct) after induction of stroke in experimental models (in this model, a reduction in GAT-3/4 expression in neurons was observed) [71]. In the motor cortex, where the stroke was induced, eGABA_A_Rs largely contain *α*_5_ and *δ* subunits. The *α*_5_ selective benzodiazepine inverse agonist L655,708 produced a significant reduction in the tonic current amplitude in peri-infarct neurons of slices from poststroke animals as well as improving the performance of animals in an *in vivo* motor task [71]. In a similar manner, the gain of function of eGABA_A_Rs in typical absence seizures provides compelling preclinical data for the development of inverse agonists selective for *α*_4_-*δ* subunit containing GABA_A_Rs which may have potential therapeutic value in this type of nonconvulsive epilepsy.
